# A digital PCR method for identifying and quantifying adulteration of meat species in raw and processed food

**DOI:** 10.1371/journal.pone.0173567

**Published:** 2017-03-20

**Authors:** Junan Ren, Tingting Deng, Wensheng Huang, Ying Chen, Yiqiang Ge

**Affiliations:** 1 College of Food Science and Nutritional Engineering, China Agricultural University, Beijing, China; 2 Agro-product Safety Research Center, Chinese Academy of Inspection and Quarantine, Beijing, China; 3 China Rural Technology Development Center, Beijing, China; University of Hyogo, JAPAN

## Abstract

Meat adulteration is a worldwide concern. In this paper, a new droplet digital PCR (ddPCR) method was developed for the quantitative determination of the presence of chicken in sheep and goat meat products. Meanwhile, a constant (multiplication factor) was introduced to transform the ratio of copy numbers to the proportion of meats. The presented ddPCR method was also proved to be more accurate (showing bias of less than 9% in the range from 5% to 80%) than real-time PCR, which has been widely used in this determination. The method exhibited good repeatability and stability in different thermal treatments and at ultra-high pressure. The relative standard deviation (RSD) values of 5% chicken content was less than 5.4% for ultra-high pressure or heat treatment. Moreover, we confirmed that different parts of meat had no effect on quantification accuracy of the ddPCR method. In contrast to real-time PCR, we examined the performance of ddPCR as a more precise, sensitive and stable analytical strategy to overcome potential problems of discrepancies in amplification efficiency discrepancy and to obtain the copy numbers directly without standard curves. The method and strategy developed in this study can be applied to quantify the presence and to confirm the absence of adulterants not only to sheep but also to other kinds of meat and meat products.

## Introduction

Adulteration of meat products occurs frequently worldwide [1–3]. The condition occurs such as deliberately adding cheaper derivatives to bulk out minced meat products, unwitting adulteration by poor manufacturing methods, or mislabeling (deliberate or unwitting) of one meat as another. In China, the sheep and goat meats are often adulterated with chicken because chicken is much less expensive than sheep or goat meat [3–5]. To protect the interests of consumers, many countries and regions have issued regulations regarding the labeling of meat products [6–8], and several methods for the identification of animal species based on DNA [9–12] or protein (peptides) [13–16] have been developed to coordinate with the implementation of those regulations. However, determining of the ratio of adulteration remains a critical issue because it is difficult to discriminate whether the adulterants were included deliberately or inadvertently.

Numerous papers have demonstrated the applicability of real-time PCR for meat quantification [17–21]. However, the copy numbers calculated using the Ct value from real-time PCR that can be affected by amplification efficiency and impurities in the DNA solution. Discrepancies from the actual copy numbers would result in greater deviation in the quantification of the weight proportion of species. Digital PCR (dPCR) is a new technique for precise quantification that can eliminate the effects of matrixes, improve the sensitivity and precision, and provide an absolute measurement of nucleic acid concentration without the use of standard curves [22]. This technique has been extensively used in several areas, including quantitative gene expression analysis [23], bacterial abundance [24], identification of genetically modified organisms [25] and food authentication [26]. In the quantification of DNA from transgenic soy, dPCR may be more suitable for quantitative analysis, as it exhibits a measurement uncertainty of only 17% or below for a single reaction [27]. However, compared with other methods, there have been fewer reports about digital PCR for the quantitative analysis of meat species.

The difficulty in the quantification of meat is transforming copy numbers to the weight proportion of meat. The DNA yields (the copies of each gene) vary, even with equal weights of different types of meat, because the cell density, genome size and the copy numbers of target genes in the genomic DNA vary among different animal species [28]. Recent research reported that there was a close linear relationship between the raw meat weight and DNA content and gene copy numbers [29]. Such an approach requires two calibration curves, which would increase the experimental complexity and the bias of the results. Additionally, the adulterant often comes from various parts of the animal, and meat products are usually treated with high temperatures or high hydrostatic pressure. To the best of our knowledge, very little work has been performed to study the robustness of quantification by ddPCR when using meat from different parts or using processed meat.

Hence, in this study, a droplet digital PCR (ddPCR) assay for the accurate and precise quantification of the chicken weight fraction in sheep or goat was developed and validated. To address the difficulty of using the copy numbers calculated by ddPCR for quantification of the weight proportions of meats, a constant (multiplication factor) was introduced. We compared the established method with real-time PCR, which is widely used in the detection of meat adulteration. Additionally, to verify the repeatability and accuracy of this method, we analyzed the influence of different parts of the chicken and different processing conditions, such as thermal and ultra-high pressure treatments. The goals of this study were to determine the fractions of chicken in sheep or goat, whether added unwittingly or deliberately, and to provide a precise method to aid law enforcement agencies in the control of food adulteration.

## Materials and methods

### Preparation of reference mixed meat samples

Authentic fresh meat (skeletal muscle) samples from pork (*Sus scrofa*), beef (*Bostaurus*), horse (*Equus caballus*), rabbit (*Oryctolagus cuniculus*), donkey (*Equus asinus*), sheep (*Ovis aries*), goat (*Capra hircus*), dog (*Canis lupus*), chicken (*Gallus gallus*), duck (*Anas platyrhynchos*), pigeon (*Columba livia*), goose (*Anser anser*), and turkey (*Meleagris gallopavo*), along with commercial sheep and goat samples, including minced mutton, meat rolls, kebabs, dumplings, lamb sausage and cumin lamb, were purchased from a local market in Beijing, China. To determine the dynamic range, repeatability and limits of quantification of the methods, different proportions of fresh chicken and sheep meat were prepared to cover a range of 1–80% chicken/sheep (160 mg _chicken_/40 mg _sheep_, 100 mg _chicken_/100 mg _sheep_, 40 mg _chicken_/160 mg_sheep_, 20 mg _chicken_/180 mg _sheep_, 10 mg _chicken_/190 mg _sheep_, 2 mg _chicken_/198 mg _sheep_).

To ensure that the extracted DNA accurately represented the proportions of different meats, 200 mg samples of the tissue mixtures described above were frozen in liquid nitrogen and ground at 25 times/s for 60 s using a TissueLyserⅡ (QIAGEN, Germany) until mixed evenly.

### DNA extraction

DNA were extracted from 200 mg of each meat sample by using a modified cetyltrimethyl ammonium bromide (CTAB) protocol (ISO 21571:2005) [30]. Following DNA extraction, the purity and concentration of the DNA solutions were measured by UV photometry with NanoDrop 1000 (Germany).

### Primers and probes

To detect sheep, goat and chicken, the housekeeping gene replication protein A1 (*RPA1*) was selected as the target detection sequence. The *RPA1* sequences of sheep, goat, and chicken were BLASTed against representative species, including *Sus scrofa* (NC 010454.3), *Bos Taurus* (AC 000176.1), *Gallus gallus* (NC 006106.3), *Anas platyrhynchos* (NW 004677611.1), *Equus caballus* (NC 009154.2), *Meleagris gallopavo* (NC 015031.2) and other distant taxa. The primers and probes for digital PCR were designed using Primer Premier 5.0, and target sequences were aligned using DNAMAN. TaqMan probes were labeled with 6-carboxyfluorescein (FAM) at the 5’ end and black hole quencher (BHQ I) at the 3’ end (Table 1).

**Table 1:** Primer and probe sequences for quantitative PCR.

Primer/Probe	Sequences (5’-3’)	NCBI Reference Sequence
Sheep-F (Goat-F)	CTGACACACGGGACACMTCTCC	NC_019468.1
Sheep-R (Goat-R)	AAGCTAAACATGGACCCACATG	
Sheep-P (Goat-P)	FAM-TAAGCCAGCCTTGTGCGTGTGGTCC-BHQ1	
Chicken-F	CAGAACCACACTCAACCTGTCTGA	NC_006106.3
Chicken-R	TCGGGGAAATGTCTTACTGCAAG	
Chicken-P	FAM-CTCCTAGCAGCCTGTGCCAAGGCCA-BHQ1	

### Droplet digital PCR assay and specificity detection

The ddPCR assays were carried out in a total volume of 20 μL containing 50 ng of DNA template, 10 μL 2 ×ddPCR Master Mix (Bio-Rad, USA), 1 μL of 10 μM primer-F, 1 μL of 10 μM primer-R, and 0.5 μL of 10 μM probes. A Bio-Rad QX200 ddPCR droplet generator (Bio-Rad, USA) was used to divide the 20 μL mixture into approximately 20,000 droplets, with the target DNA segments and PCR reagents being randomly distributed into the droplets. The primer and probe sequences are shown in Table 1. The thermal parameters were as follows: 10 min at 95°C, 30 s at 94°C, followed by 50 cycles of 30 s at 94°C and 1 min at 60°C, followed by enzyme inactivation at 98°C for 10 min and holding at 4°C. Finally, the amplified products were analyzed using a QX200 droplet reader (Bio-Rad, USA).

After the PCR assays were finished, the copy numbers were automatically analyzed using QuantaSoft Version 1.6.6 (Bio-Rad, USA). The absolute copy numbers per panel were estimated according to the number of positive wells and the total number of partitions. According to the Poisson distribution, the copy number per droplet was calculated using the following equation:

$$M=\text{-}\ln(1-\frac{P}{R})$$ (1)

Where the R value was the total number of separated droplets and P was the number of wells positive for the *RPA1* gene [31].

To test the specificity of the sheep, goat and chicken ddPCR reactions, 200 mg of each of 13 types of fresh meat (sheep, goat, pork, chicken, duck, goose, pigeon, beef, horse, dog, rabbit, turkey and donkey) was frozen and ground. DNA was extracted using the CTAB protocol mentioned above. Approximately 50 ng of DNA was used for each ddPCR assay.

### Quantification strategy based on multiplication factor

Because there is a discrepancy between the DNA fraction and the mass proportion of meats, to avoid deviation, it is necessary to correct the concentration of species-specific genes quantified by digital PCR by using multiplication factors. The copy numbers of chicken and sheep in the DNA solution were related to the weight of mixture used for DNA extraction and the copy numbers of the chicken and sheep per unit of mass. Hence, to yield a sample with the specific type of meat, Eq 2 was used:

$$M=\frac{Q}{C},$$ (2)

where M is the mass in weight of specific meat in the sample, Q is the copy number of the *RPA1* gene in the DNA solution as calculated by ddPCR measurement, and C is the *RPA1* gene copy number of unit mass.

Since the ratios of mass of chicken and sheep could help us obtained the chicken proportion, we should calculate the ratios of the mass of chicken and sheep. So we deduced Eq 3:

$$\frac{Mc}{Ms}=\frac{\frac{Qc}{Cc}}{\frac{Qs}{Cs}}=\frac{Cs}{Cc}\times \frac{Qc}{Qs},$$ (3)

where Mc and Ms are the masses of chicken and sheep, respectively; Qc and Qs are the copy numbers of chicken and sheep in the mixtures, respectively; and Cc and Cs represent the copy numbers per unit mass of chicken and sheep, respectively.

Under a fixed procedure for the extraction of DNA (grinding, protease and denaturant), for a mixed meat product with a type of processing and tissue composition, the multiplication factor *k* (Cs/Cc) could be considered invariable. Therefore,

$$\frac{Mc}{Ms}=k\times \frac{Qc}{Qs}.$$ (4)

To determine the *k* value, five mixtures of different proportions of sheep and chicken (1%, 10%, 20%, 50% and 80% chicken (w/w)) underwent ddPCR quantification of the concentration of the species-specific *RPA1* gene. Each sample was tested three times in parallel.

To verify the accuracy and stability under a fixed level of quality, six parallel mixtures of 50% (w/w) sheep and chicken were prepared in order to quantify the copy numbers of the *RPA1* genes from chicken and sheep. The *k* value and RSD were calculated.

### Real-time PCR quantification approaches

Real-time PCR was performed using an Applied Biosystems 7500 real-time PCR system (Applied Biosystems, USA) in 25 μL reaction mixtures, which consisted of 12.5 μL of 2× TaqMan Gene Expression Master Mix (Applied Biosystems, USA), 1.0 μL of each primer (10 μM), 0.5 μL of probe (10 μM), 5 μL of template DNA (20 ng/μL and 5 μL of ddH_2_O. PCR was initiated at 50°C for 2 min, 95°C for 10 min, followed by 40 cycles at 95°C for 15 s and 60°C for 60 s. Every round of the experiment was performed on a single 96-well reaction plate to minimize variability due to thermal cycling.

To simplify the experimental procedure and reduce the complexity of the conversion from copy numbers to the weight of meat, equal weights of sheep and chicken were mixed as a standard sample. To correct the discrepancy of copy numbers of equal weights of chicken and sheep, the equal virtual concentration, 1×10^5^ copies/μL, was assumed to represent the copies of *RPA1* for sheep and chicken in the standard sample DNA solution. Six 4-fold dilutions of a standard sample DNA solution were measured in triplicate. By plotting mean C_T_ vs. log(DNA copy numbers), standard curves for both species were constructed.

For unbiased comparisons between the two quantification methods, mixtures with chicken levels of 80%, 50%, 20%, 10%, 5%, and 1% were selected for real-time PCR assay and ddPCR. The C_T_ values recorded by sheep and chicken detectors were directly interpolated on the standard curve to yield the concentration of species-specific genes. The proportion of chicken meat in the sample was expressed as:

$$P_{\mathrm{chicken}}=100\times C_{\mathrm{chicken}}/(C_{\mathrm{chicken}}+C_{\mathrm{sheep}}),$$ (5)

where C_chicken_ and C_sheep_ were the copy numbers obtained from the standard curve of sheep and chicken detectors, respectively.

### Dynamic range, limit of quantification (LOQ), and limit of detection (LOD)

The squared regression coefficient (R^2^) of the linear regression line between the actual content of adulterant (x-axis) and the measured values of the meat fraction (y-axis) was used to evaluate the dynamic range of the quantification of mixed meat.

The LOQ was determined by evaluating the relative standard deviation (RSD) and bias of meat mixtures ranging from 1%-10%. Three replicates of ddPCR tests were run for each level.

The chicken in the binary (refer the mixture used) was accordingly diluted to 1%, 0.8%, 0.5%, 0.2% and 0.1% for sensitivity detection. Each level of concentration was analyzed in three replicates.

### Repeatability and accuracy

To determine the repeatability and accuracy of the method for detection of adulterant ingredients, six replicates of ddPCR tests were run for each mixture, with chicken levels of 10%, 5%, 4%, 3%, 2% and 1% (w/w), by different operators in our lab. In order to minimize the impact of other factors, the operators started with extracted DNA.

### Robustness of the method

The texture, processing (heat treatment or ripening) and tissue ratios of the meat fractions used for the production of mixed meat products may influence the results produced by methods that measure the content of DNA, such as quantitative PCR [32]. To evaluate the effect of different body parts of chicken on quantification, the meat from chicken legs, breasts and wings were mixed with sheep in two proportions (5% and 50%), treated with liquid nitrogen, and ground in a TissueLyser II (QIAGEN). The measured values were corrected by a *k* value, and the deviation of two levels (5% and 50%) of mixtures tested by ddPCR was calculated.

The effects of thermal treatment with different temperatures and times on the quantitative method were investigated. The binary mixtures of chicken and sheep in two proportions (5% and 50%) were treated in a refrigerator at -18°C for 24 h or at 4°C for 12 h, respectively. In addition, binary mixtures were steamed at 50°C, 80°C, or 100°C in a digital circulation water bath (Memmert), and thermally processed at 120°C in an autoclave (Tuttnauer 3850 EL) for 10 minutes and 20 minutes, respectively.

Further, the influence of different intensities of ultra-high pressure on the accuracy of quantification was also considered. Both of the two proportions (5% and 50%) of meat mixtures were processed at 200 MPa, 300 MPa, 400 MPa, 500 MPa and 600 MPa for 10 minutes and 20 minutes by 650-5L HHP (high hydrostatic pressure, HHP, KeFa). The processed mixtures were ground, and the DNA was extracted and tested in the same way as the fresh samples. Then, the copy numbers measured by ddPCR were converted to the proportion of meat by the *k* value, which were deduced theoretically and verified by experiments as described in the “*Quantification strategy based on multiplication factor*” section.

## Results and discussion

### Determination and verification of multiplication factors

The ratios of the copy numbers of unit mass of chicken and sheep in five proportions (1%, 10%, 20%, 50% and 80% chicken) are shown in Table 2. The average value of the *k* value for five different meat fractions was 0.8, and the relative standard deviation (RSD) was 9.9%, demonstrating that the *k* values were stable against any mass ratio of chicken and sheep.

**Table 2:** The ratios of copy numbers of unit mass with different proportions of chicken.

Chicken proportion	Three parallel tests of the concentration of chicken (copies/μL)			Three parallel tests of the concentration of sheep(copies/μL)			K	Means	RSD
80%	770	769	768	160	159	160	0.8	0.8	9.9%
50%	375	378	376	292	295	305	0.8		
20%	163	157	161	484	472	476	0.7		
10%	103	100	99	714	718	721	0.8		
1%	9.7	9.1	7.6	556	536	547	0.8		

To verify the accuracy and stability of the *k* value, equal masses of muscle were used to obtain the multiplication factor, which depicted the difference in target copy numbers between the two species. To determine the *k* value, six parallel references mixtures (50_chicken_/50_sheep_) were measured by ddPCR. The average copy numbers for sheep in the mixture were 265 copies/μL, 336 copies/μL, 324 copies/μL, 373.3 copies/μL, 379.3 copies/μL, and 327 copies/μL, while the average copy numbers for chicken in the mixture were 335.33 copies/μL, 406 copies/μL, 395.3 copies/μL, 471.7 copies/μL, 480 copies/μL, and 384.7 copies/μL. The *k* value was approximately 0.8 (RSD = 3.1%), which could be used to convert copies of target DNA extracted from lean mixtures to the weight proportions of each species (S1 Table).

These results suggest that the *k* value is always approximately 0.8, being independent of the relative content of chicken and sheep. Therefore, the *k* value can be applied to correct for the proportion of meat. Compared to previously reported ddPCR methods, which require the production of a matrix-adapted series of proportional materials encompassing the whole range of expected meat proportions [29], the use of a single-point equal mass proportion material in ddPCR-based food assays in the present work simplifies the experimental step for species quantification without producing standard curves.

### Comparison of ddPCR and RT-PCR for quantification

The following formula (4) was used: Mc/Ms = k×(Qc/Qs), k = 0.8, Mc/Ms = 0.8×(Qc/Qs).The measured chicken fractions in the meat mixtures (80%, 50%, 20%, 10%, 5%, and 1% w/w) were 79.1% (RSD = 0.4%), 49.8% (RSD = 1.3%), 20.8% (RSD = 1.9%), 9.9% (RSD = 2.0%), 4.8% (RSD = 0.5%), and 1.3% (RSD = 13.8%), respectively (Table 3).

**Table 3:** Comparison of the limit of quantification for sheep and chicken mixtures (weight/weight) by ddPCR and real-time PCR.

Actual value (%)	Measured value (%)		RSD (%)		Bias (%)	
	ddPCR	qPCR	ddPCR	qPCR	ddPCR	qPCR
80	79.1±0.3	68.1±0.1	0.4	2.5	-1.2	-14.9
50	49.8±0.7	34.9±0.1	1.3	4.8	-0.4	-30.2
20	20.8±0.4	12.7±0.1	1.9	1.9	4.2	-36.5
10	9.9±0.2	5.4±0.1	2.0	9.1	-1.0	-45.8
5	4.8±0.1	2.9±0.1	0.5	8.7	-3.4	-41.1
1	1.3±0.2	0.6±0.1	13.8	8.6	24.8	-35.8

Actual value (%): Chicken content is expressed as the actual percentage.

Measured value (%): The average content of chicken as measured by ddPCR and real-time PCR.

Bias (%): Bias of the average content of chicken measured by ddPCR and real-time PCR compared with the actual value.

**Fig 1:**
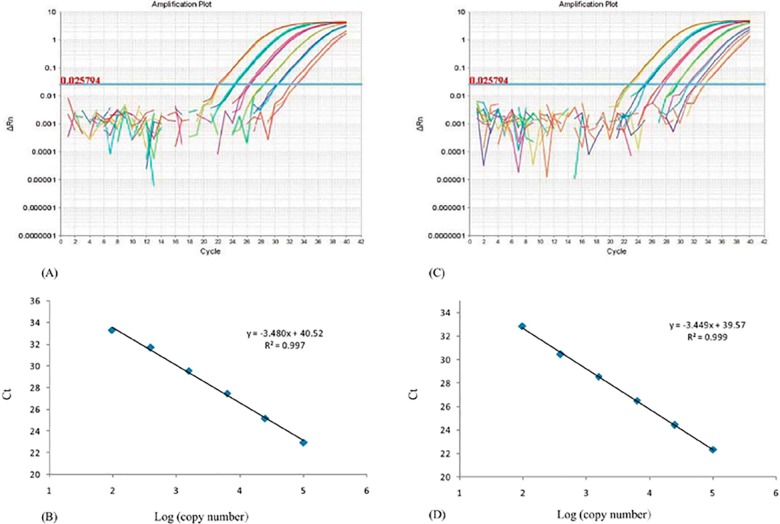
Amplification plots and standard curves for the RPA1 genes of chicken and sheep. (A) Amplification plots of 4-fold dilution series of binary mixture DNA of chicken (from 1×10^5^ to 9.7×10^1^ copies/μL). (B) Linearity test, regression line parameters of 4-fold dilution series of chicken DNA (from 1×10^5^ to 9.7×10^1^ copies/μL) as standards. (C) Amplification plots of 4-fold dilution series of binary mixture DNA of sheep (from 1×10^5^ to 9.7×10^1^ copies/μL). (D) Linearity test, regression line parameters of 4-fold dilution series of binary mixture DNA of sheep (from 1×10^5^ to 9.7×10^1^ copies/μL) as standards. Each data point represents the mean of three replicates.

The real-time PCR method utilizes a linear relationship between the Ct value and the logarithm of initial template copy numbers in reactions, which is assumed to be proportional to the weight of chicken and sheep in the mixtures. In this work, two standard curves were established for the quantification of chicken and sheep. The amplification efficiencies for chicken and sheep in meat mixtures were 94.96% and 93.80%, respectively. In addition, from the standard curve generated with Ct values against log (copy numbers of chicken) or log (copy numbers of sheep), the equations were established as follows: Ct = - 3.449log (copy numbers of chicken) + 39.57 (R^2^ = 0.999); Ct = - 3.480 log (copy numbers of sheep) + 40.52 (R^2^ = 0.997) (Fig 1). These two equations could be used to estimate the chicken and sheep contents in a mixture; thus, the fraction of chicken in sheep (w/w) could be determined based on the copy ratio of chicken and sheep.

**Fig 2:**
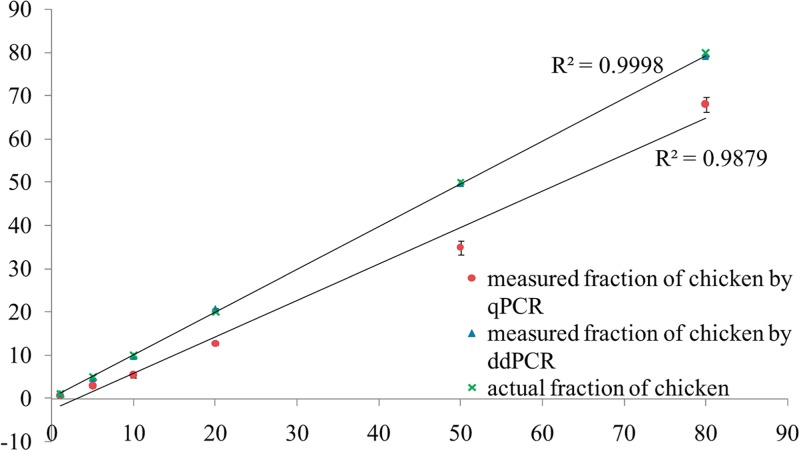
Dynamic range of the ddPCR assay for quantification of chicken and sheep fractions. The vertical axis represents the measured fraction of chicken in sheep (w/w) by ddPCR and qPCR. The horizontal axis shows the actual fraction of chicken in sheep (w/w). Three replicates for each data point were analyzed. The points indicate the concentrations of 80%, 50%, 20%, 10%, 5% and 1% (w/w) of chicken in sheep. Linearity between the actual chicken fraction (w/w) and the measured chicken fraction (w/w) by ddPCR. The correlation coefficient (R^2^) for the weight of chicken was 0.9998 for ddPCR and 0.9878 for real-time PCR.

According to the results obtained from the same mixtures analyzed with the two methods shown in Table 3, both the ddPCR and qPCR methods had a relative standard diversion (RSD) ≤ 25% for quantification and showed good repeatability. However, the bias of qPCR was greater than 25%, while that of ddPCR was less than 5% for chicken mixtures ranging from 5% to 50%. The deviation of 80% chicken was -14.94% with real-time PCR, whereas the bias was -1.15% with ddPCR. The results showed that the difference reached 44.80% for the 10% chicken fraction. According to Fig 2, the correlation coefficient (R^2^) for the actual chicken fraction in a mixture of chicken and sheep was 0.9879 based on real-time PCR. The accuracy of qPCR quantification was lower than that of ddPCR.

Digital PCR is an absolute quantification technique used to calculate the copy number without a standard curve and Ct, thereby avoiding the discrepancy produced by differing amplification efficiencies of different samples. The bias of 1% chicken samples measured by real-time PCR was -35.8%, whereas it was 24.8% when measured using ddPCR. Compared to a previous study, the bias of real-time PCR for 1% sample is 65%. Overall, the ddPCR method is more accurate than real-time PCR in meat adulteration quantification [32].

Real-time PCR has been widely used in the quantification of nucleic acids in various species, as demonstrated with a large range of adulteration concentrations (experimentally from 1% to 80%). Compared to real-time PCR, ddPCR is much more accurate and easier to perform; accordingly, it requires much more costly equipment and consumables [33].

### Dynamic range, limit of quantification (LOQ) and limit of detection (LOD)

The measurement of the proportion of chicken in sheep, the slope of the standard curve, and the correlation coefficient (R^2^) of the respective species in meat mixtures are summarized in Fig 2. The lower amounts that decreased to 1% w/w still showed a good recovery. The results showed that the deviation was less than 2% when the chicken concentration ranged from 5% to 80%, and the quantification method presented in our study could provide an accurate analysis with low RSD (Table 3). The chicken proportion of the dynamic range of sheep was between 1% and 80%.

The LOQ was defined as 1% chicken in sheep, which could be precisely quantified with an RSD less than 25% following the FAO Guidelines on the performance criteria and validation of methods for the detection, identification and quantification of specific DNA sequences and specific proteins in food [34] (Table 4). In contrast, in another quantification study based on the ddPCR technique, which used a two-step conversion that increased the bias of quantification, the LOQ was 10 mg (10%) for pork, and the bias was approximately 17% [29]. In our study, the deviation of 10% (w/w) was approximately less than ±8.17%, and the LOQ was defined as 1%. Because digital PCR is an extremely precise quantification technique [31,35,36], it can be used to help law enforcement agencies and testing organizations to ascertain whether or not adulteration is intentional. The 1% lowest level of quantification of adulterant is achieved with routine testing and food quality control.

**Table 4:** LOQ, repeatability and accuracy test.

Meat mixtures^2^	A^1^									B									The RSD of different operators
	Six parallels of quantification						Mean Value (%)	RSD (%)	Bias (%)	Six parallels of quantification						Mean Value (%)	RSD (%)	Bias (%)	
10%	9.22	9.26	9.03	9.19	9.39	9.01	9.18±0.14	1.56	-8.17	9.24	9.49	9.01	9.14	9.25	9.84	9.33±0.30	3.18	-6.72	2.54%
5%	5.08	4.75	4.74	5.03	4.87	5.31	4.97±0.22	4.45	-0.70	5.19	5.20	4.89	4.80	4.77	5.04	4.98±0.19	3.85	-0.35	3.97%
4%	4.24	3.72	3.85	4.1	3.80	4.23	3.99±0.23	5.75	-0.24	4.23	4.32	3.87	3.75	4.10	4.11	4.06±0.21	5.34	1.55	5.37%
3%	2.72	3.02	2.89	2.70	2.82	3.12	2.88±0.17	5.74	-4.08	2.79	2.91	3.07	2.72	2.86	2.80	2.86±0.12	4.28	-4.71	4.85%
2%	2.14	2.22	2.10	2.13	2.33	2.11	2.17±0.09	4.08	8.60	2.34	2.11	2.28	2.12	2.29	2.27	2.24±0.10	4.26	11.81	4.26%
1%	1.30	1.32	1.14	1.13	1.08	1.36	1.22±0.11	9.71	22.10	1.15	1.34	1.28	1.12	1.23	1.20	1.21±0.08	7.23	21.04	8.19%

^1^A and B represents the different operators in our lab. ^2^Meat mixtures indicate the chicken weight percentage of the sheep content.

The limit of detection (LOD) was defined as the lowest percentage of chicken content that could be reliably detected. To determine the LOD of our method, mixtures containing various percentages, ranging from 0.1% to 1% were prepared. The sensitivity levels observed are shown in S2 Table. The results showed that the positive wells could be stably seen even in the 0.1% group. Thus the LOD of the system reached 0.1% chicken content in sheep.

### Specificity, repeatability and accuracy

**Fig 3:**
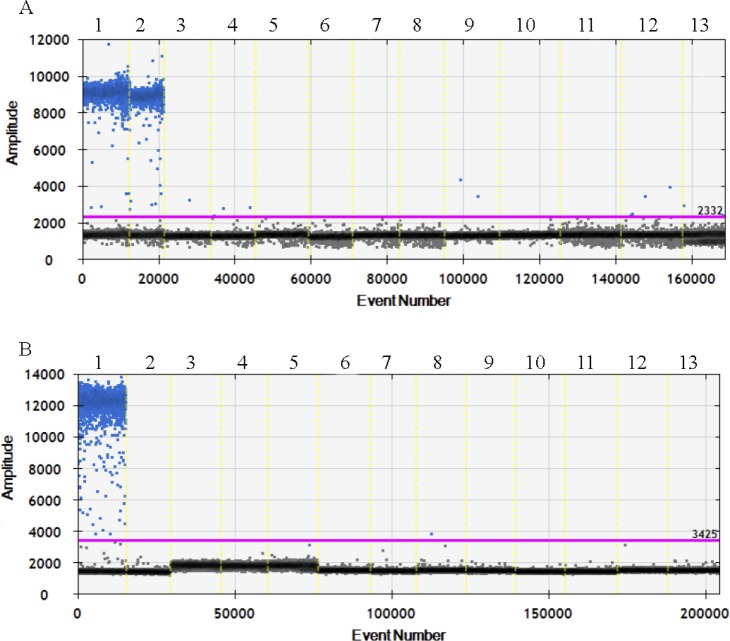
The specificity results of primers and probes of chicken, sheep and goat. The horizontal axis indicates the event number of 13 kinds of meats. The vertical axis indicates the amplitude of samples. (A) Upper frame: droplet cluster positive for FAM (sheep, goat and other meats). Lower frame: droplet cluster negative for FAM (sheep, goat and any other meats) negative droplet cluster. Lanes: 1, sheep; 2, goat; 3, pork; 4, beef; 5, horse; 6, rabbit; 7, donkey; 8, dog; 9, chicken; 10, duck; 11, pigeon; 12, goose; 13, turkey. (B) Upper region: droplet cluster positive for FAM (chicken and any other meats). Lower region: droplet cluster negative for FAM (chicken and any other meats) negative droplet cluster. Lanes: 1, chicken; 2, sheep; 3, goat; 4, beef; 5, horse; 6, rabbit; 7, donkey; 8, dog; 9, chicken; 10, duck; 11, pigeon; 12, goose; 13, turkey.

Specificity is the crucial precondition for a successful qualitative and quantitative method in food authentication. To determine the specificity of the sheep and chicken ddPCR reaction, meats from 13 animal species were tested: sheep, goat, chicken, pork, duck, goose, pigeon, beef, horse, dog, rabbit, turkey and donkey. Six varieties of chicken, five varieties of sheep and three varieties of goat were amplified to sequencing, the results showed that the amplified region is conservation for our research (S1 Fig). The two pairs of species-specific primers and probes only amplified target sequences from sheep, goat and chicken, respectively, and they showed no cross-reaction with any non-target species. These results confirmed that the method was capable of identifying chicken mixed with or substituted for sheep and goat specifically (Fig 3).

Intra-laboratory repeatability validation was performed by two different experienced operators to determine the chicken content of the same sample on two different days. Variability of≤10% was observed in each of the six cartridges for the determination of the chicken and sheep fractions by two operators (Table 4). The RSD values in the 1% to 10% chicken mixtures were less than 9.71%. The deviations in 2% and 10% chicken mixtures were less than 12%, and that from the 1% chicken mixtures was less than 22.10%. These results demonstrate that meat fractions can be quantitated repeatedly and accurately with this assay.

### Robustness of the method

#### Effects of different parts of the animal species

To verify whether the repeatability and accuracy of quantification in the adulteration assay change with the body location, we selected three different parts of chicken (legs, breasts and wings) to make a meat mixture for testing. The results showed that the bias of the measured value in three locations of chicken mixed in sheep is less than ±9%, and the RSD values were all under 6% (S3 Table). We concluded that meats located in different parts of the body did not significantly affect the quantitative results in this method.

Mitochondrial DNA has been extensively used for PCR templates in species determination [37–40] due to its high copy numbers. However, nuclear genomic DNA is better for meat quantification because its constant haploid copy number makes the concentration of genes per gram of meat relevant to the quantity of meat tested. Thus, it is more predictive than mitochondrial markers, which vary sharply in the copy numbers in different organs and individuals [41,42]. According to a previous report, there is a five-fold inter-tissue variation in mtDNA content per cell that results in either an underestimation (-70%) or overestimation (+160%) of DNA content [26]. In the present study, the nuclear genomic gene *RPA1* was used for meat quantification, it is not necessary to determine which parts of meat came from.

#### Effect of the treatment temperature

**Fig 4:**
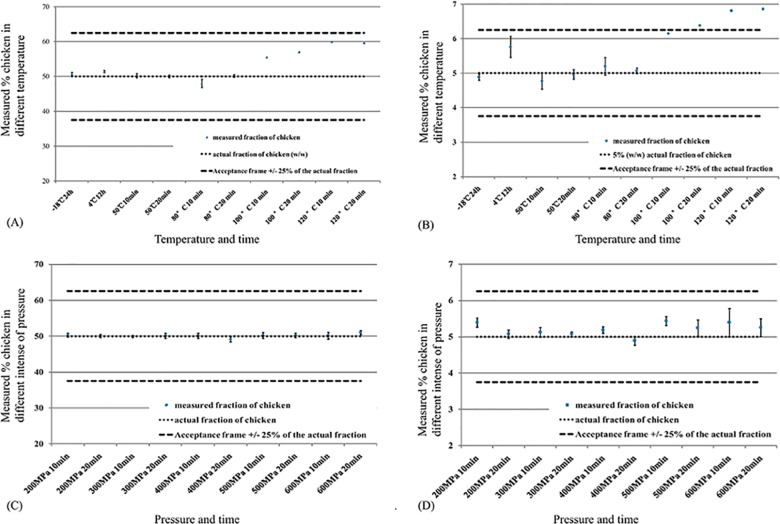
The repeatability results of 50% and 5% chicken in sheep processed under different temperature and pressure conditions for different durations. The target chicken fraction is indicated by a dotted line. The acceptance criterion for repeatability is ±25% of the target proportion, represented by the dashed lines. Error bars represent the standard deviation between the replicates for each treated condition. (**A**) The 50% chicken fraction processed at different temperatures for different durations and measured by ddPCR (three replicates in ten conditions). (**B**) The 5% chicken fraction processed at different temperatures for different durations and measured by ddPCR (three replicates in ten conditions). (**C**) The 50% chicken fraction processed at different pressure intensities and for different durations and measured by ddPCR (three replicates in ten conditions). (**D**) The 5% chicken fraction processed at different pressure intensities and for different durations and measured by ddPCR (three replicates in ten conditions).

To demonstrate the robustness of the ddPCR assay, different temperature treatments were implemented, including -18°C for 24 h; 4°C for 12 h; 50°C, 80°C, 100°C and 120°C for 10 min and 20 min (S4 Table). Using the precision analysis, the results showed that the RSD values of all samples, with the temperature ranging from -18°C to 120°C, were less than 10%. This illustrated that the method in the present study maintained a very high precision and stability. Bias in the measurement of 50% and 5% chicken fraction samples processed under 100°C varied in the ranges of ±5% and ±15%, respectively. The method maintained a high level of accuracy for samples treated at -18°C to 80°C. Bias in the measurement of mixtures containing 50% and 5% chicken became larger with higher temperatures and longer treatments. When the temperature increased from 100°C to 120°C, for 50% mixtures, the errors increased from +10% to +20% (Fig 4A), and for 5% chicken, the errors increased from +22% to +37% (Fig 4B), respectively. This suggested that 100°C is the turning point for accuracy in the measurement of heat-treated samples. The ddPCR quantification method established here is more accurate when mixtures were treated at temperatures less than 100°C, and the accuracy decreases significantly with the length of processing. In addition, we found that the deviation had a positive relationship with the extension of time and the increase of temperature when treated above 100°C.

It has been suggested that the loss of accuracy is probably due to the different degradation rates of DNA from different species, and it will increase with the temperature of heat processing [43,44]. The loss of accuracy may also be caused by the recovery rate of genomic DNA in extractions, since protein-protein and protein-DNA (polysaccharides) interactions that occur during heat treatment lead to discrepancies in DNA release in extraction [45]. Both events induced variations of the *k* value, which may explain the positive increase in deviation in synchronization with the increase of temperature from 100°C to 120°C. Therefore, it is recommended that the reference meat mixture be treated at the same treatment for calculating the multiplication factor.

#### Effect of ultra-high pressure (UHP) processing

In the meat industry, ultra-high pressure is mainly used to increase shelf life and improve the food safety of ready-to-eat meat products as a novel post-packaging non-thermal decontamination technology. Therefore, UHP has been employed in the meat industry for the past two decades [46]. The effect of ultra-high pressure on the quantification of meat mixtures is summarized in S5 Table. The RSD values and bias were less than 25% in 50% and 5% chicken fraction mixtures subjected to treatment with pressure from 200 MPa to 600 MPa (Fig 4C and 4D). High pressure can destroy the tertiary structure and quaternary structure of proteins but has little effect on covalent bonds. Heating causes DNA degradation and may influence the efficiency of PCR; in contrast, treatment with high pressure at room temperature does not damage the primary structure of DNA. The precision and accuracy of quantitative determination are not correlated with treatment with high hydrostatic pressure in the range of 200–600 MPa. In general, high pressure has no significant effect on the quantification accuracy of meat mixtures.

#### Testing of commercial samples

To demonstrate the applicability of the ddPCR assay, 14 commercial sheep and goat samples from local supermarkets were analyzed by ddPCR and real-time PCR. Table 5 indicated that 5 samples were positive for chicken DNA by ddPCR, and one brand of lamb sausage was positive for ddPCR, but negative for real-time PCR. These results demonstrated that the sensitivity of the developed ddPCR method was better than that of real-time PCR. Dumplings, the other lamb sausage and a brand of kebab were positive for chicken according to both ddPCR and real-time PCR, showing adulteration with chicken meat at concentrations from 30% to 75%. However, the RSD values of ddPCR method were lower than those of real-time PCR, indicating that ddPCR showed higher precision and accuracy. The percentage of chicken content in cumin lamb was lower than the LOQ (1%); thus, we presumed that the sample might have been contaminated unintentionally. These results showed that this developed technology can be utilized for commercially available meat products and that it shows better sensitivity, precision and accuracy than real-time PCR.

**Table 5:** Identification and quantification of chicken in commercial goat/sheep products.

	Sample	Identification		Quantification	
		ddPCR	Real-time PCR	ddPCR	Real-time PCR
Fresh meat	Minced mutton	Negative	Negative	0.0	0.0
	Meat rolls 1	Negative	Negative	0.0	0.0
	Meat rolls 2	Negative	Negative	0.0	0.0
	Meat rolls 3	Negative	Negative	0.0	0.0
	Meat rolls 4	Negative	Negative	0.0	0.0
	Kebab 1	Positive	Positive	39.04±0.59	40±5.38
	Kebab 2	Negative	Negative	0.0	0.0
	Kebab 3	Negative	Negative	0.0	0.0
	Kebab 4	Negative	Negative	0.0	0.0
	Kebab 5	Negative	Negative	0.0	0.0
	Dumplings	Positive	Positive	58.57±1.20	55.47±5.56
Meat products	Lamb sausage 1	Positive	Positive	73.92±1.86	76.64±7.29
	Lamb sausage 2	Positive	Negative	0.12±5.44	—
	Cumin lamb 1	Positive	Positive	0.65±2.98	1.23±8.98

Meat rolls 1–4 represent four brands of sheep meat rolls, respectively.

Kebabs 1–5 represent five different brands of kebab, respectively.

Lamb sausages 1, 2 represent two different brands of lamb sausage, respectively.

## Conclusion

In this work, we showed that the ddPCR method is a novel, accurate, reliable and easy quantification strategy for the determination of animal species in fresh and processed meat products. Normally, the copy numbers cannot be converted to proportions directly because the quantities of the haploid genome per gram of meat and the densities of cells are different in meats from different species. The strategy that we tested was to introduce a constant (multiplication factor) deduced theoretically to transform the ratio of copy numbers to the proportion of meats. This quantification method can be reliably used down to the 1% (w/w) level for chicken and sheep mixtures, and the LOD is 0.1% (w/w). Compared to real-time PCR, the bias of the chicken proportion in the range from 5% to 80% was less than 9% according to this assay. Moreover, it was concluded that the part of chicken meat had no effect on the accuracy of quantification. Further, this research also showed that a high level of accuracy was maintained under different pressure and temperature conditions. This method, which introduces a constant to transform the ratio of copy numbers to the proportion of meats, has the potential to be applied in routine assays for quantification of food adulteration, not only in raw or processed meat products, but also in many other applied fields in which this information is needed.

## Supporting information

S1 Fig The multi-alignments of *RPA1* gene target sequences. The multi-alignments of (A) *RPA1* gene target sequences from Beijing fatty chicken and other varieties, (B) *RPA1* gene target sequences from sheep, goat and other varieties.
(DOCX)

S1 Table The unit mass of chicken and sheep copy numbers and ratios. (DOCX)

S2 Table The sensitivity for detecting chicken in sheep (LOD). (DOCX)

S3 Table The repeatability and accuracy of measurements of proportions of chicken and sheep when using meats from different parts of the chicken carcass. (DOCX)

S4 Table The repeatability and accuracy of measurements of the proportions of chicken and sheep when processed at different temperatures for different durations. (DOCX)

S5 Table The repeatability and accuracy of measurements of the proportions of chicken and sheep when processed under different intensities of pressure. (DOCX)
