# Supplementary figures and images for: A digital PCR method for identifying and quantifying adulteration of meat species in raw and processed food
Source: PLoS One. 2017 Mar 20;12(3):e0173567. doi: 10.1371/journal.pone.0173567 (PMC5358868; doi:10.1371/journal.pone.0173567)

S1 Fig

(A)

**
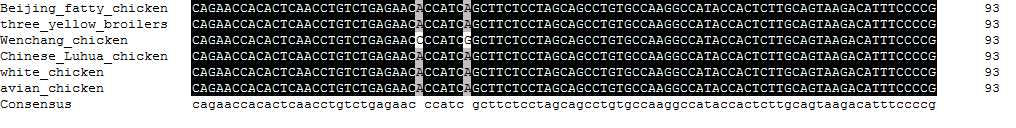
**

(B)
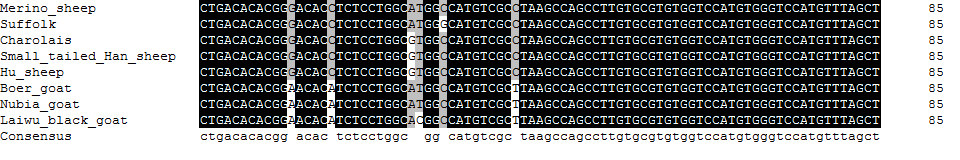

Supplement: S1 Fig — The multi-alignments of (A) RPA1 gene target sequences from Beijing fatty chicken and other varieties, (B) RPA1 gene target sequences from sheep, goat and other varieties. (DOCX) [file pone.0173567.s001.docx]
