# Supplementary material for: A digital PCR method for identifying and quantifying adulteration of meat species in raw and processed food
Source: PLoS One. 2017 Mar 20;12(3):e0173567. doi: 10.1371/journal.pone.0173567 (PMC5358868; doi:10.1371/journal.pone.0173567)
Supplement: S1 Table — (DOCX) [file pone.0173567.s002.docx]

**S1 Table** The unit mass of chicken and sheep copy numbers and ratios.

| Samples | Concentration of sheep  (copies/μL) | Concentration of chicken  (copies/μL) | *K* | Means | RSD |
| --- | --- | --- | --- | --- | --- |
| 1 | 265 | 335.3 | 0.8 | 0.8 | 3.1% |
| 2 | 336 | 406 | 0.8 |  |  |
| 3 | 324 | 395.3 | 0.8 |  |  |
| 4 | 373.3 | 471.7 | 0.8 |  |  |
| 5 | 379.3 | 480 | 0.8 |  |  |
| 6 | 327 | 384.7 | 0.9 |  |  |
