# Supplementary material for: A digital PCR method for identifying and quantifying adulteration of meat species in raw and processed food
Source: PLoS One. 2017 Mar 20;12(3):e0173567. doi: 10.1371/journal.pone.0173567 (PMC5358868; doi:10.1371/journal.pone.0173567)
Supplement: S2 Table — (DOCX) [file pone.0173567.s003.docx]

**S2 Table** The sensitivity of chicken in sheep (LOD).

| Meat mixtures | Three parallels of chicken(copies/μL) | | | RSD |
| --- | --- | --- | --- | --- |
| 1% | 18 | 21 | 25 | 16.46% |
| 0.8% | 11 | 10 | 17 | 29.89% |
| 0.5% | 5 | 3 | 3 | 31.49% |
| 0.2% | 4 | 3 | 2 | 33.33% |
| 0.1% | 2 | 2 | 1 | 34.64% |
