# Supplementary material for: A digital PCR method for identifying and quantifying adulteration of meat species in raw and processed food
Source: PLoS One. 2017 Mar 20;12(3):e0173567. doi: 10.1371/journal.pone.0173567 (PMC5358868; doi:10.1371/journal.pone.0173567)
Supplement: S3 Table — (DOCX) [file pone.0173567.s004.docx]

**S3 Table** The repeatability and accuracy of measurements of proportion of chicken/sheep with chickens from different parts of chicken carcass.

| location | ratio | Average (%) | Bias (%) | RSD (%) |
| --- | --- | --- | --- | --- |
| Leg | 50% | 52.5±0.0 | 4.9 | 3.6 |
|  | 5% | 5.2±0.0 | 3.2 | 2.8 |
| Breast | 50% | 46.4±0.0 | -7.2 | 5.5 |
|  | 5% | 4.7±0.2 | -6.6 | 4.5 |
| Wing | 50% | 48.3±0.0 | -8.7 | 0.4 |
|  | 5% | 4.9±0.2 | -3.0 | 4.1 |
