# Supplementary material for: A digital PCR method for identifying and quantifying adulteration of meat species in raw and processed food
Source: PLoS One. 2017 Mar 20;12(3):e0173567. doi: 10.1371/journal.pone.0173567 (PMC5358868; doi:10.1371/journal.pone.0173567)
Supplement: S4 Table — (DOCX) [file pone.0173567.s005.docx]

**S4 Table** The repeatability and accuracy of measurements of proportion of chicken/sheep processed at different temperature for different time.

| Treatment condition | Meat mixtures (%) | Mean value (%) | RSD (%) | Bias (%) |
| --- | --- | --- | --- | --- |
| -18 °C 24h  4 °C 12h  50°C 10 min | 50% | 50.8±0.4 | 0.8 | 1.5 |
|  | 5%  50% | 4.9±0.1  51.4±0.3 | 1.9  0.7 | -2.3 |
|  |  |  |  | 2.8 |
|  | 5%  50% | 5.8±0.3  50.2±0.6 | 5.4  1.2 | 15.3 |
|  |  |  |  | 0.4 |
|  | 5% | 4.8±0.2 | 4.9 | -4.7 |
| 50 °C 20 min  80°C 10 min | 50% | 50.1±0.4 | 0.8 | 0.1 |
|  | 5%  50% | 5.0±0.1  48.1±1.2 | 2.8  2.6 | -0.7 |
|  |  |  |  | -3.9 |
|  | 5% | 5.2±0.3 | 4.8 | 4.2 |
| 80°C 20 min | 50% | 50.2±0.4 | 0.8 | 0.3 |
|  | 5% | 5.1±0.1 | 1.7 | 13.8 |
| 100 °C 10 min | 50% | 55.5±0.1 | 2.6 | 10.9 |
|  | 5% | 6.2±0.1 | 9.9 | 22.9 |
| 100 °C 20 min | 50% | 57.0±0.1 | 0.6 | 14.1 |
|  | 5% | 6.4±0.2 | 5.4 | 27.6 |
| 120 °C 10 min | 50% | 59.9±0.1 | 0.3 | 19.9 |
|  | 5% | 6.8±0.1 | 2.6 | 36.2 |
| 120 °C 20 min | 50% | 59.6±0.2 | 0.8 | 19.2 |
|  | 5% | 6.9±0.1 | 1.2 | 37.1 |
