# Supplementary material for: A digital PCR method for identifying and quantifying adulteration of meat species in raw and processed food
Source: PLoS One. 2017 Mar 20;12(3):e0173567. doi: 10.1371/journal.pone.0173567 (PMC5358868; doi:10.1371/journal.pone.0173567)
Supplement: S5 Table — (DOCX) [file pone.0173567.s006.docx]

**S5 Table** The repeatability and accuracy of measurements of proportion of chicken/sheep processed in different intensity of pressure

| Treatment condition | Meat mixtures (%) | Mean value (%) | RSD (%) | Bias (%) |
| --- | --- | --- | --- | --- |
| 200MPa 10min | 50% | 50.3±0.5 | 1.0 | 0.6 |
|  | 5% | 5.4±0.1 | 2.3 | 7.9 |
| 200MPa 20min | 50% | 50.1±0.5 | 1.0 | 0.1 |
|  | 5% | 5.1±0.1 | 2.3 | 1.5 |
| 300MPa 10min | 50% | 49.9±0.3 | 0.6 | -0.2 |
|  | 5% | 5.1±0.1 | 2.6 | 2.5 |
| 300MPa 20min | 50% | 50.1±0.7 | 1.4 | 0.3 |
|  | 5% | 5.1±0.1 | 1.4 | 1.5 |
| 400MPa 10min | 50% | 50.2±0.7 | 1.4 | 0.3 |
|  | 5% | 5.2±0.1 | 1.7 | 3.7 |
| 400MPa 20min | 50% | 49.2±0.7 | 1.4 | -1.6 |
|  | 5% | 4.9±0.1 | 2.4 | -2.2 |
| 500MPa 10min | 50% | 50.2±0.8 | 1.7 | 0.4 |
|  | 5% | 5.4±0.1 | 2.3 | 8.7 |
| 500MPa 20min | 50% | 50.2±0.6 | 1.3 | 0.5 |
|  | 5% | 5.2±0.2 | 4.3 | 4.9 |
| 600MPa 10min | 50% | 50.1±1.0 | 1.9 | 0.3 |
|  | 5% | 5.4±0.4 | 7.2 | 8.0 |
| 600MPa 20min | 50% | 51.0±0.5 | 1.1 | 2.1 |
|  | 5% | 5.3±0.3 | 4.7 | 5.1 |
